# Possible underdiagnosis of ulcerative colitis in tertiary care can affect medication use and access to healthcare facilities

**DOI:** 10.3389/fimmu.2026.1693891

**Published:** 2026-02-10

**Authors:** Sara Ferraro, Claudia Bartolini, Irma Convertino, Lorenzo Bertani, Francesco Costa, Emiliano Cappello, Olga Paoletti, Sabrina Giometto, Rosa Gini, Ersilia Lucenteforte, Marco Tuccori

**Affiliations:** 1 Unit of Pharmacology and Pharmacovigilance, Department of Clinical and Experimental Medicine, University of Pisa, Pisa, Italy; 2 Osservatorio di Epidemiologia, Agenzia Regionale di Sanità della Toscana, Florence, Italy; 3 Department of Internal Medicine, Tuscany North-West Azienda Sanitaria Locale (ASL), Pontedera Hospital, Pontedera, PI, Italy; 4 Department of Translational Research and New Technologies in Medicine and Surgery, University of Pisa, Pisa, Italy; 5 IBD Unit, Department of General Surgery and Gastroenterology, Pisa University Hospital, Pisa, Italy; 6 Unit of Medical Statistics, Department of Clinical and Experimental Medicine, University of Pisa, Pisa, Italy; 7 Department of Statistics, Computer Science and Applications “G. Parenti”, University of Florence, Florence, Italy; 8 Department of Diagnostics and Public Health, University of Verona, Verona, Italy

**Keywords:** administrative databases, drug-utilization, missed diagnosis, tertiary care, ulcerative colitis

## Abstract

**Background:**

Ulcerative colitis (UC) is a chronic inflammatory bowel disease where diagnostic delays can worsen the clinical outcomes and increase the strain on healthcare systems. This study investigated the frequency of potentially missed UC diagnoses in tertiary care and their impact on treatment patterns and healthcare utilization.

**Methods:**

We conducted a retrospective cohort study using Tuscany’s regional healthcare database (2006–2020). Adults newly diagnosed with UC between 2011 and 2018 were included. A “possible missed diagnosis” was defined as a hospital or emergency department (ED) visit for gastrointestinal (GI) symptoms occurring 7–60 months before the UC diagnosis. We assessed the initiation of azathioprine and non-conventional therapies, as well as the rates of ED visits, hospital admissions, and surgery. Survival analyses and Cox regression models were applied.

**Results:**

Among 3,804 patients with UC, 313 (8.3%) had prior GI-related tertiary care visits suggestive of a missed diagnosis. The mean time to diagnosis was 27.5 months. Compared with those who were timely diagnosed, these patients were not more likely to start azathioprine or non-conventional therapies. However, subjects with a possible missed diagnosis had higher rates of ED visits [adjusted hazard ratio (aHR) = 1.8, 95%CI = 1.5–2.0], hospitalizations (aHR = 1.4, 95%CI = 1.2–1.7), and combined urgent care encounters (aHR = 1.5, 95%CI = 1.3–1.7) compared with other patients.

**Conclusions:**

Patients with a potentially missed UC diagnosis are more likely to need emergency and inpatient care, despite receiving similar treatments. Promoting earlier recognition of UC symptoms in tertiary care may reduce avoidable hospital use and improve disease management.

## 1 Introduction

Ulcerative colitis (UC) is an inflammatory bowel disease (IBD) that causes inflammation and ulcers of the column and rectum. The incidence of UC is increasing worldwide, and the highest incidence and prevalence have been reported in Northern Europe, USA, Canada, and Australia (1). Within Europe, there are differences in the incidence of UC, with western and northern countries having higher incidences than eastern and southern countries (2).

UC develops most often in the second or third decade of life (3, 4), although a bimodal age distribution of incidence has been described, with the main onset peak between 15 and 30 years and a second smaller peak between ages 50 and 70 years (5). The main serious complications of UC are hospitalization, access to the emergency department (ED), or UC-related surgery (6). In the management of UC, the primary goal is to induce and maintain remission of the disease, with the long-term aim of preventing disability, colectomy, and colorectal cancer. Conventional therapy with 5-aminosalicylic acid is the standard first-line pharmacological treatment (7). When progression occurs, patients can be treated with several therapeutic options such as immunosuppressive agents, including azathioprine or advanced therapy options, e.g., biological drugs; however, even surgery could be necessary in the most refractory patients.

A timely identification of the disease is crucial to treat patients early and to avoid rapid disease progression. A diagnostic delay is a well-known phenomenon occurring in IBD, and it is observed more in patients with Crohn’s disease than in those with UC (8). However, a diagnostic delay in UC is often associated with several complications, including severe disease progression, poor response to therapy, the need for surgery, and a lower quality of life (9–11). Common symptoms of UC are both specific (e.g., rectal bleeding) and generic (e.g., abdominal pain, diarrhea, and weight loss). Notably, almost one-third of patients have silent illness; thus, diagnosis can be challenging, particularly in the early phases of the disease. Moreover, there is poor knowledge of UC in non-specialist gastroenterologists, including general practitioners and the general population, leading to underestimation. For these reasons, missing diagnoses or underdiagnoses can occur in tertiary care, resulting in possible diagnostic delays and causing negative impacts on the disease progression of patients.

This study aimed to investigate possible missing diagnoses of UC in the tertiary care setting using healthcare administrative databases. Firstly, we identified UC subjects with a hospital access for gastrointestinal (GI) clinical manifestations suggestive of UC, but which occurred before an actual UC diagnosis (possible missing diagnosis). Secondly, we assessed the time elapsed from the earliest hospital access with UC symptoms to the UC diagnosis. Finally, we evaluated the impact of these possible missing diagnoses and of the time to diagnosis on medication use and access to healthcare facilities.

## 2 Materials and methods

We conducted a retrospective observational study with a cohort design on data extracted from the administrative healthcare database of Tuscany (Italy), a region with approximately 3.6 million inhabitants. This paper was written according to the Strengthening the Reporting of Observational Studies in Epidemiology (STROBE) guidelines (see **Supplementary Data** for the STROBE checklist).

### 2.1 Data source

The Italian population is covered by a national, single-payer, universal, public health system. The regional healthcare administrative database includes services and drugs supplied to patients (12) and comprises data electronically collected since 2004. In particular, for this study, we extracted data recorded between January 1, 2006, and December 31, 2020, from the following repositories: the drug-dispensing registry, the co-payment exemption registry, and the registry of hospital discharge records and ED admissions. The drug-dispensing registry collects detailed records of the drug prescriptions dispensed by both community and hospital pharmacies. It includes specific information such as the active substance name, the Anatomical Therapeutic Chemical (ATC) code, the Italian marketing authorization code, the prescribed dose, the pharmaceutical formulation, and the exact date of dispensing. The co-payment exemption registry includes records of subjects with a code of exemption from co-payment for specific healthcare services. These exemptions are granted based on specific disease conditions (including UC) or socioeconomic criteria.

The hospital discharge registry systematically collects data on hospital admissions and discharges. It records the primary and secondary diagnoses, as well as the medical procedures performed during hospitalization, all coded according to the International Classification of Diseases, 9th edition (ICD-9). In addition, it includes the dates of hospital admissions and discharges.

The ED admissions registry compiles records of all ED visits. It provides detailed information on the diagnosis at the time of the visit, coded using ICD-9, along with the exact date of ED admission.

### 2.2 Study population

The UC cohort included patients with a first ever record of UC diagnosis OR a first ever record of co-payment exemption code for UC from June 1, 2011, to December 31, 2018. UC diagnosis was defined by the first ever UC record retrieved in the hospital discharge registry or in the ED admission registry OR the first ever record of exemption from co-payment for UC. The ICD-9 and exemption codes are listed in Box 1 of the tables in the **Supplementary Material**. The index date (ID) identified the patient entry date in the cohort. ID was defined as the date of the first record of UC or the date of the first record of co-payment exemption for UC, whichever came first, and we considered this date as a proxy indicator of the date of UC diagnosis. The follow-up period was 2 years after the ID. We excluded patients: 1) with less than 5 years of data in the look-back period (i.e., period before the ID); 2) who were younger than 18 years at the ID; 3) with less than 2 years of follow-up (observation period after ID); 4) with a record of a cancer-related disease exemption code (048) in the 5-year look-back period, i.e., the time window preceding the ID during which the exclusion criteria were assessed; and 5) with a record of UC diagnosis OR a record of co-payment exemption code for UC in the look-back period. Censorings were defined by the end of follow-up, death, or patient loss to follow-up, whichever came first.

### 2.3 Data analysis

Exposure was defined as the occurrence of a possible missed diagnosis of UC. A possible missed diagnosis was identified by the earliest access to ED or hospitalization for GI events in the look-back period, representing a proxy of UC symptoms (ICD-9 codes) (Box 2 of the tables in the **Supplementary Material**) (11). We considered these symptoms to be moderate to severe as they require evaluation in tertiary care settings. Patients were classified as exposed if they had a potential missed diagnosis recorded within the period 7–60 months prior to the ID. Exposed patients were further stratified into two subgroups: those with a missed diagnosis occurring between 7 and 18 months before the ID (short-term missed diagnosis) and those with a missed diagnosis recorded between 19 and 60 months before the ID (long-term missed diagnosis). Non-exposed patients included both individuals without any recorded missed diagnosis during the entire look-back period and those with a missed diagnosis occurring within the 6 months preceding the ID. The 6-month time window was considered part of the ongoing diagnostic process.

We performed two different analyses. In the first analysis, we evaluated the impact of a possible missed diagnosis on different outcomes by comparing non-exposed and exposed patients (dichotomous variables). In the sub-analysis, we assessed the same outcomes by comparing the non-exposed patients to both patients with a short-term missed diagnosis and those with a long-term missed diagnosis (categorical variables). The outcomes were drug utilization and healthcare facility use. For the drug utilization outcomes, we assessed the time-free survival from the first dispensation of azathioprine and the first dispensation or infusion of non-conventional therapy drugs, i.e., infliximab, adalimumab, golimumab, vedolizumab, ustekinumab, and tofacitinib (the drugs and specialist procedure codes are listed in Boxes 3 and 4 of the tables in the **Supplementary Material**). For the outcomes related to the use of healthcare facilities, we assessed the time-free survival from the first event of: surgery for UC (Box 5 of the tables in the **Supplementary Material**), ED access for any cause, hospitalization for any cause, and first ED access OR hospitalization for any cause. The following covariates were included in the model: age at ID, gender, and number of concomitant drugs at cohort entry. Finally, we assessed the mean and the distribution of gastroenterological visits (Box 6 of the tables in the **Supplementary Material**) by calculating the mean number of visits; the cumulative number of visits after 1 and 2 years of follow-up; and the number of patients with zero to three visits, with four to six visits, with seven to 10 visits, and those with more than 10 visits. All analyses were conducted for both dichotomous and categorical variables.

### 2.4 Statistical analysis

We first tabulated the distributions of the dichotomous and categorical variables within the study population to perform the first and second descriptive analyses. Differences among the patient categories for these variables were evaluated using the chi-squared test at a significance level of *α* = 0.05. For continuous variables, we computed the mean and standard deviation (SD) of the time elapsed from the occurrence of an ED access or hospitalization for GI causes to the ID. Homoscedasticity was assessed using the Bartlett test, while normality was evaluated via the Shapiro–Wilk test. When these assumptions were met, differences between groups were examined using analysis of variance (ANOVA); otherwise, the non-parametric Kruskal–Wallis rank-sum test was employed. All statistical tests were conducted at an *α* level of 0.05.

For the drug utilization analysis, we estimated the Kaplan–Meier time-free survival curve from the ID to the first: dispensation of azathioprine, dispensation of non-conventional therapies, and infusions of non-conventional therapy. Events that occurred at the ID were not included in the analysis. We compared the curves using the log-rank test at 12 and 24 months. Subsequently, the Cox regression model adjusted for the covariates age, sex, and number of concomitant drugs at cohort entry was used to estimate the hazard ratio (HR) and corresponding 95% confidence interval (CI) for the drug utilization outcomes. Patients without a possible missed diagnosis represented the reference category. In the analysis of healthcare facility use, we estimated the Kaplan–Meier time-free survival curve from the ID to the first: ED access for any cause, hospitalization for any cause, and ED access OR hospitalization for any cause (whichever came first). The survival analysis and the HR estimation for these outcomes were conducted following the same methodology as that for the drug utilization analysis.

## 3 Results

**Figure 1:**
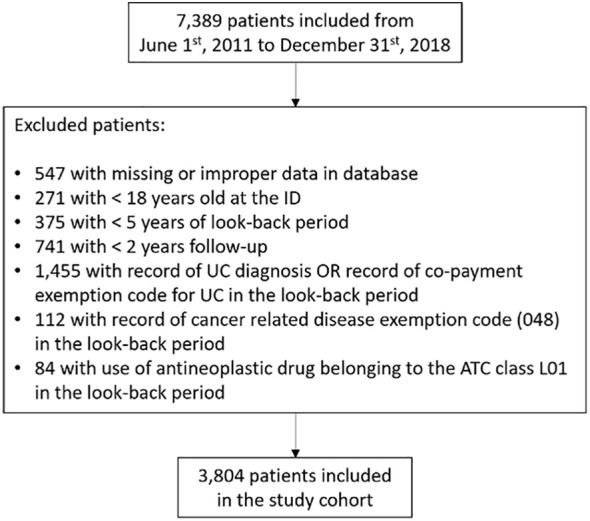
Flowchart of the included patients.

According to the inclusion and exclusion criteria, the study cohort included 3,804 patients (the flowchart of the included patients is displayed in (**Figure 1**). In particular, 2,168 (57%) and 1,636 (43%) patients were included due to a diagnosis of UC in the hospital discharge records or the ED admission records and a disease exemption for UC, respectively. There were 1,645 (47.1%) women, and the mean age was approximately 53 years, with 0.31 (SD = 1.0) concomitant drugs. Details of the included patients are reported in **Supplementary Table S1**. The first analysis showed that 313 (8.3%) patients had hospital access for GI causes before the ID, i.e., a possible missing diagnosis. The sub-analysis revealed that 113 (3%) patients had a short-term possible missed diagnosis and that 200 (5.3%) subjects had a long-term possible missed diagnosis (**Table 1**). The mean time to diagnosis was 27.5 (SD = 16.1) months.

**Table 1:** Characteristics of patients according to the occurrence of a possible missed ulcerative colitis diagnosis.

Patients	No missed diagnosis (reference) (n = 3,491)	Possible missed diagnosis (n = 313)	P-value	Short-term possible missed diagnosis (n = 200)	Long-term possible missed diagnosis (n = 113)	P-value
Age (years), mean (SD)	53.5 (18.6)	52.0 (19.2)	0.0149	53.25 (19.3)	51.31 (19.2)	0.222
Women, *n* (%)	1,645 (47.1)	158 (50.5)	0.0030	60 (53.1)	98 (49.0)	0.409
Concomitant drugs, mean (SD)	0.31 (1.0)	0.42 (1.2)	0.035	0.4 (1.3)	0.42 (1.1)	0.052

SD, standard deviation.

**Figure 2:**
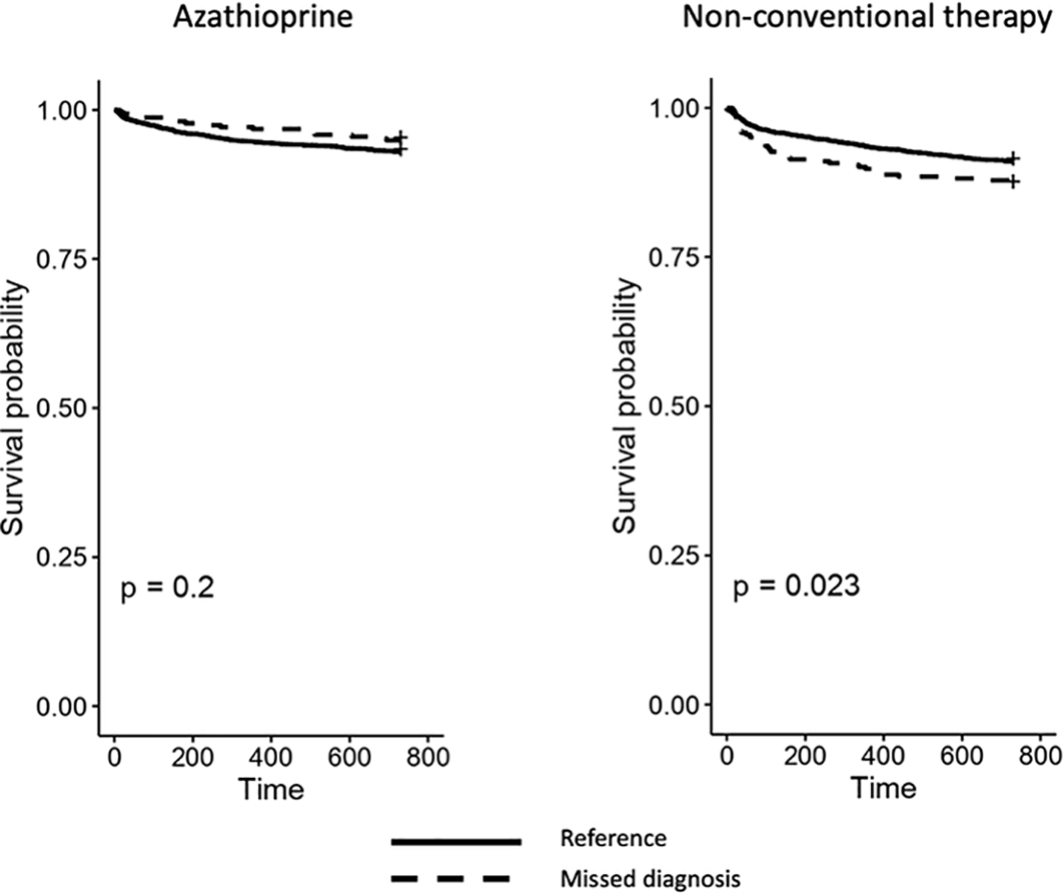
Kaplan–Meier curves for azathioprine and non-conventional therapy outcomes computed for the dichotomous variables of missed diagnosis. The graphics describe the time-free survival from the first dispensation of azathioprine and non-conventional therapy for the dichotomous variable of potential missed diagnosis. The figure shows that patients with a possible missed diagnosis have a higher probability of receiving a biologic drug than patients without in the follow-up period.

**Figure 3:**
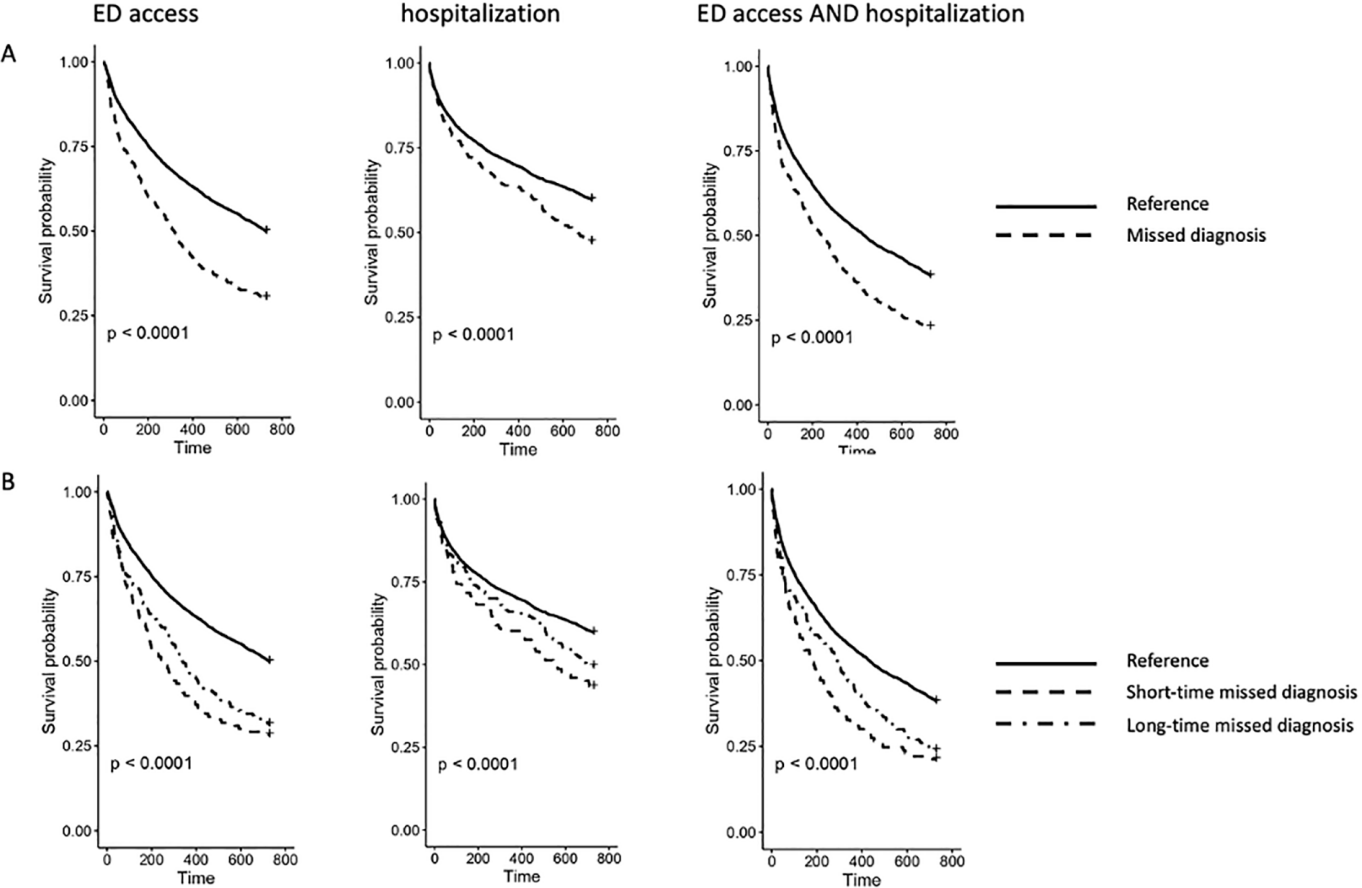
Kaplan–Meier curves for emergency department (ED) access, hospitalization and ED access, AND hospitalizations computed for the dichotomous and categorical variables of missed diagnosis. The graphics describe the time-free survival from the first ED access or hospitalization for any cause, the first ED access for any cause, and the first hospitalization for any cause. The figure shows that patients with a possible missed diagnosis have a higher probability of developing the observed events than patients without for both the dichotomous **(A)** and categorical **(B)** variables of missed diagnosis.

The second analysis showed that azathioprine and a non-conventional therapy drug were dispensed to 261 and 354 patients, respectively. The estimated HR revealed no significant risks observed for the drug utilization outcomes for either the dichotomous or the categorical variables (**Table 2**). In accordance, the Kaplan–Meier curves of the time-free survival from azathioprine and non-conventional therapy for both dichotomous and categorical variables are displayed in **Figure 2** and **Supplementary Figure S1**. The HR values obtained for the analysis of the healthcare facility use outcomes are displayed in **Table 3**. It was found that 2,401 (63.1%) patients had at least one ED access or hospitalization for any cause in the follow-up period. In the primary analysis, we observed that the adjusted hazard ratios (aHRs) for surgery (1.9, 95%CI = 0.4–8.3), ED access (1.8, 95%CI = 1.5–2.0), hospitalization (1.4, 95%CI = 1.2–1.7), and the composite outcome (1.5, 95%CI = 1.3–1.7) showed a higher risk of these events for patients with a possible missed diagnosis compared to those without. In the sub-analysis, we observed a significant risk of access to ED or hospitalization (composite outcome) for any cause in both patients with a short-term missed diagnosis (aHR = 1.7, 95%CI = 1.4–2.0) and those with a long-term missed diagnosis (aHR = 1.4, 95%CI = 1.2–1.7) compared to patients without. No significant aHR was found for surgery. The Kaplan–Meier curves showed a significantly lower probability to survive free from all the analyzed events for patients with a possible missed diagnosis than those without, except for surgery (**Figure 3** and **Supplementary Figure S2**). The sub-analysis confirmed the findings, indicating that this risk could not be dependent from the length of time between the occurrence of UC symptoms and the diagnosis of UC (**Figure 3**).

**Table 2:** Adjusted hazard ratio for the drug utilization outcomes estimated for the dichotomous and categorical variables of possible missed ulcerative colitis diagnosis.

Therapy	No missed diagnosis, *n*	Possible missed diagnosis, *n*	aHR (95%CI)	Short-term possible missed diagnosis, *n*	aHR (95%CI)	Long-term possible missed diagnosis, *n*	aHR (95%CI)
Azathioprine	245	16	0.8 (0.4–1.1)	4	0.5 (0.2–1.3)	12	0.7 (0.4–1.3)
Non-conventional therapy^a^	314	40	1.4 (1.0–1.9)	14	0.5 (0.9–2.5)	26	1.3 (0.9–2.0)

*CI*, confidence interval; *aHR*, adjusted hazard ratio.

a Dispensations+infusions, adjusted for age, gender, and number of concomitant drugs.

**Table 3:** Adjusted hazard ratio for the healthcare facility use outcomes estimated for the dichotomous and categorical variables of possible missed ulcerative colitis diagnosis.

Event	No missed diagnosis, *n*	Possible missed diagnosis, *n*	aHR (95%CI)	Short-term possible missed diagnosis, *n*	aHR (95%CI)	Long-term possible missed diagnosis (*n*)	aHR (95%CI)
Surgery	13	2	1.9 (0.4–8.3)	0	0 (0–Inf)	2	1.1 (0.8–13.5)
ED access^a^ or hospitalization^a^	2,160	241	1.5 (1.3–1.7)	89	1.7 (1.4–2.0)	152	1.4 (1.2–1.7)
Hospitalization^a^	1,406	165	1.4 (1.2–1.7)	64	1.6 (1.2–1.9)	101	1.3 (1.1–1.6)
ED access^a^	1,750	218	1.8 (1.5–2.0)	81	1.9 (1.6–2.4)	137	1.7 (1.4–2.0)

*CI*, confidence interval; *aHR*, adjusted hazard ratio.

a For any cause, adjusted for age, gender, and number of concomitant drugs.

**Table 4** shows the distribution of GI visits. The mean number of visits for patients without a missed diagnosis was 2.7 (SD = 4.4), while it was 3.2 (SD = 4.4) for those with a possible missed diagnosis. Patients with a short-term missed diagnosis showed a higher mean number of visits than patients with a long-term missed diagnosis. In the 2-year follow-up, the number of visits ranged from 0 to 3 for the majority of patients with and without a missed diagnosis.

**Table 4:** Distribution of gastrointestinal visits according to stratification for possible missed ulcerative colitis diagnosis.

	Patients				
	Overall (*n* = 3,804)	No missed diagnosis (*n* = 3,491)	Possible missed diagnosis (*n* = 313)	Short-term possible missed diagnosis (*n* = 113)	Long-term possible missed diagnosis (*n* = 200)
Visits, mean (SD)	2.8 (4.4)	2.7 (4.4)	3.2 (4.4)	3.8 (5.0)	2.8 (3.9)
Cumulative no. of visits at follow-up					
1 year	1,687	1,542	145	60	85
2 years	1,067	941	126	49	77
Patients, *n* (%)					
0–3 visits	3,614 (95)	3,321 (95.1)	293 (93.6)	106 (93.8)	187 (93.5)
4–6 visits	112 (2.9)	100 (2.9)	12 (3.8)	3 (2.6)	9 (4.5)
7–10 visits	35 (0.9)	33 (0.9)	2 (0.6)	1 (0.9)	1 (0.5)
>10 visits	43 (1.1)	37 (1.1)	6 (1.9)	3 (2.7)	3 (1.5)

SD, standard deviation.

## 4 Discussion

This study aimed to evaluate the impact of potential missed diagnoses of UC within a tertiary care setting on both the pharmacological management and the utilization of healthcare resources. According to the literature, diagnostic delay can be used as a marker for missed diagnoses of UC, making this subgroup an ideal benchmark for comparing the findings of this study. The type of data source and the research methodology greatly influence the ability of a study to identify missed diagnoses. Our study was exclusively conducted on an administrative database, while the most commonly used data sources for investigation of a diagnostic delay in UC include registries (13, 14), medical charts (15), and information obtained from interviews or questionnaires submitted to patients with UC (16). Administrative claim databases, such as those used in our investigation, are particularly effective at capturing missed diagnoses within tertiary care settings. In contrast, alternative sources such as disease registries or survey-based studies are capable of recognizing missed diagnoses even in primary care environments or when symptoms are relatively mild. Consequently, these differing approaches can yield significantly divergent results. In addition, an interview-based approach is affected by the ability of patients to recall relevant clinical details of past events. Moreover, questionnaires can be created with different questions and communication strategies, which could contribute to the scarce comparability of the results of the available studies.

Literature evidence shows that the mean time to diagnosis is extremely variable, and it could be explained mainly by the heterogeneity of the different studies. In particular, each study provided a different definition of diagnostic delay using specific signs and symptoms for the identification of UC diagnosis. Moreover, differences in healthcare systems, socioeconomic settings, and medical education contribute to the heterogeneity of the results. The present study revealed that 8.3% of patients with UC had a possible missed diagnosis within the tertiary care setting, with a mean time of 27.5 months from GI symptoms to diagnosis. We found that 91.7% of patients likely had a timely diagnosis as they were diagnosed within 6 months from early symptoms captured in healthcare administrative databases or they did not access the ED or hospital at all. Instead, patients with short-term and long-term possible missed diagnosis comprised 3% and 5.3% of the whole cohort, respectively. However, the majority of authors did not differentiate between a timely diagnosis and a diagnostic delay and did not categorize the delay as short or long. However, they were limited to assessing the time to diagnosis, likely considering a few days to diagnosis as a delay in the diagnosis. Of note is that this time usually contributes to the assessment of the mean diagnostic delay, which could have been underestimated from some authors. In our study, we considered patients diagnosed within 6 months from symptoms as timely diagnosed patients in order to overcome this possible methodological flaw. This resulted in a mean time to diagnosis longer than those reported in other studies that did not define a minimum time window that can be considered a timely diagnosis. Cantoro et al. performed a retrospective cohort study including patients with UC recorded in the registry of four Italian IBD referral centers. This cohort included 1,855 patients with UC and evaluated IBDs and the risk factors of long diagnostic delays. The median time from the onset of symptoms to the diagnosis of UC was 2 months, and approximately 72% of patients were diagnosed within 6 months, while approximately 28% of patients had a longer time to UC diagnosis (17). Similarly, Romberg-Camps et al. conducted a prospective cohort study including 600 patients with UC from an IBD registry that prospectively collected the data of both hospitalized patients and outpatients, which used medical charts to enrich the information. The median time to diagnosis was 3 months, and 88% of patients were found to be diagnosed within 1 year from the first symptoms, while 10% had a diagnostic delay up to 5 years (18).

Interview-based studies represent a method design with many limitations due to the variability of the questions and the patients’ ability to remember past events. A number of studies collected data from large study populations, which also identified both severe and not severe patients. Dubinsky et al. (19) and Lönnfors et al. (20) performed interviews with different data sources using an online research database and by sending questionnaires to members of an IBD association, respectively. However, these studies included 2,100 and 1,541 patients, respectively, showing that 47% and approximately 54% of patients were diagnosed within 1 year from the onset of symptoms, which are percentages lower than those found in the present study. Moreover, Dubinsky et al. showed a mean time to diagnosis of 24 months (19), a result similar to that of the present study. Interviews are often intended for outpatients, resulting in a higher delay in the diagnosis of UC. Vavricka et al. performed an interview-based retrospective cohort study using healthcare databases. A total of 625 patients were recruited from hospitals and private practice. This study showed that patients diagnosed within the first 4 months from the onset of symptoms comprised 50% (21). Although we considered a time window of 0–6 months for patients without a possible missed diagnosis, the percentage we found is almost double when compared with that found by Vavricka et al., probably because we did not include patients from private practice. Moreover, this study showed that patients diagnosed within the first year comprised 75% (21), which is consistent with Molander et al. who included 508 patients, members of an UC association, showing that approximately 72% of patients were diagnosed within 1 year from the first symptoms. However, this survey found a mean time to diagnosis of 27.6 months (22), which is consistent with the results of the present study. On the other hand, Zaharie et al. included 682 patients from university medical IBD referral centers only. These patients were recruited from an IBD registry, and it was demonstrated that 75% of patients were diagnosed within the first 3 months from the onset of symptoms (23).

In our research, the use of a 60-month period to define potential diagnostic delay is supported by the findings of the IMPACT survey, which was conducted under the auspices of the European Federation of Crohn’s and Colitis Associations (EFCCA) among European patients. This comprehensive online survey, which included 4,990 individuals diagnosed with IBD, revealed that 20% of participants experienced a delay of up to 5 years from the onset of symptoms to a definitive diagnosis (10).

The key finding of this study is that missed diagnoses of UC have an impact on the use of healthcare facilities. We observed that the possible occurrence of a missed diagnosis did not strongly affect the initiation of pharmacological therapies. However, the increased rates of ED visits and hospitalizations suggest that diagnostic delays might exacerbate the clinical burden and resource utilization among patients with UC. When considering drug utilization, our study showed that the time from the presumed diagnosis to the first dispensation of azathioprine or non-conventional therapies did not differ significantly between patients with and without a missed diagnosis. The significant Kaplan–Meier results obtained for non-conventional therapy indicate that there is a difference in the event-free survival over time between the groups. However, the non-significant HR suggests that the average instantaneous risk difference between groups does not reach statistical significance, possibly due to a risk of the event not constant over time, leading HR to not capture the time-specific differences that the Kaplan–Meier analysis detects. As azathioprine is not recommended as a first-line therapy for patients with newly diagnosed UC and is generally introduced to maintain remission in corticodependent patients, its use can provide a proxy for disease progression within a step-up treatment approach (7). In the literature, we found one study that evaluated the impact of diagnostic delay on azathioprine use: Walker et al. demonstrated that, excluding emergently diagnosed patients, there is no difference in the use of immunomodulators between patients with a diagnostic delay and those with a timely diagnosis in the first year after UC diagnosis (*p* = 0.117) (13). On the other hand, four studies described the impact of a delayed diagnosis of UC on non-conventional therapy and found different results. Two of these showed that advanced therapy use is more frequent in patients with a long diagnostic delay (14, 15). In particular, Szántó et al. found that biologic therapy is more common in patients with a diagnostic delay longer than 1 year (14), while Kang et al. demonstrated that a diagnostic delay >2 years is associated with the use of anti-tumor necrosis factor (TNF) drugs (15). In contrast, the other two studies did not observe a significant impact of diagnostic delay on therapy (9, 24). Lee et al. observed that the time interval from diagnosis to anti-TNF administration is not different between delayed and non-delayed patients (9). Likewise, Walker et al. observed no differences in the administration of advanced therapy between patients with a timely diagnosis and those with a non-emergently delayed diagnosis (24).

Biologic agents have revolutionized the management of moderate-to-severe UC by targeting specific inflammatory pathways. Despite their efficacy, the timing of initiation appears to be crucial. In our study, we observed that a possible missed diagnosis did not significantly impact on the treatment with non-conventional therapies. This might be attributable to the fact that the treatment algorithms for UC are standardized once a diagnosis is established, irrespective of the diagnostic delay. However, the increased rate of ED visits and hospitalizations among patients with a possible missed diagnosis may reflect a higher clinical burden and perhaps a more challenging disease course in the period leading up to the diagnosis. Therefore, ensuring a timely diagnosis is critical not only for optimizing the long-term outcomes but also for reducing the immediate strain on healthcare systems.

Although our study did not find a statistically significant association between missed diagnosis and early surgery, the heightened frequency of ED visits and hospitalizations suggests that the impact of delayed diagnoses could focus predominantly on acute disease exacerbations and the subsequent need for urgent healthcare services rather than on the irreversible anatomical changes. Three studies from the literature evaluated the impact of diagnostic delay on surgery. They found consistent evidence showing that a long diagnostic delay could increase the risk of early surgery in patients with UC.Fare clic o toccare qui per immettere il testo (9, 14, 24).Fare clic o toccare qui per immettere il testo. In this case, the results were statistically significant as the observation period was longer than that of our studies, allowing capturing more events.

A diagnostic delay is a clinical issue observed in both UC and Crohn’s disease, but is more common in patients with Crohn’s disease compared to those with UC (21). In a previous study (25) conducted using the same data source as the present research and with the same aim, we extracted a cohort of 3,342 patients with Crohn’s disease with a follow-up period of 3 years. The mean time to diagnosis was 27.2 months, and patients with a possible missed diagnosis comprised 17.5%. In particular, in patients with Crohn’s disease, 6.6% and 10.9% had short- and long-term missed diagnoses, respectively. Therefore, the frequency of a missed diagnosis in Crohn’s disease is double that observed in UC, and this result is consistent with evidence in the literature (21, 26). Unlike those with UC, patients with Crohn’s disease with a possible missed diagnosis were found to have a statistically significant risk of receiving a biologic drug compared with patients who were timely diagnosed. When comparing the use of healthcare resources, both UC and Crohn’s disease patients with a possible missed diagnosis showed a statistically significant risk of hospitalization and ED access than patients without a timely diagnosis, regardless of the length of time to diagnosis.

This study presents several limitations alongside notable strengths. Firstly, the presumed date of diagnosis may lag behind the actual diagnosis due to the inherent nature of administrative data. Patients might receive a diagnosis and be recorded much later, i.e., when they access a healthcare facility or obtain a drug dispensation. To mitigate this issue, we accounted for a 6-month lag period when defining a missed diagnosis. Secondly, we also used records of co-payment exemption for UC to assemble our study cohort. The use of exemption codes might lead to underestimation as exemptions can be granted for various reasons (such as age, disease, or economic status) without prioritization. Thirdly, misclassification could have occurred based on the GI symptoms selected to identify a potential missed diagnosis. Given the low specificity of these initial symptoms, it is possible that some events were not actually early manifestations of UC. Nonetheless, the selected symptoms were derived from GI events commonly reported as clinical manifestations of UC in several studies and guidelines. Finally, our administrative database has not been specifically validated for identifying patients with UC. Nevertheless, Italian validation studies that employed the same parameters as our inclusion criteria have yielded highly effective algorithms for the extraction of UC cases. Furthermore, our study successfully mirrored the key findings on diagnostic delay in UC as reported in the literature, although our analysis was based on administrative databases. This achievement highlights the robustness of our methodology and supports the validity of using such data sources for epidemiological research. Moreover, the direct comparison between patients with Crohn’s disease and those with UC yielded results that are consistent with established scientific evidence. Data collection was limited to the year 2020 due to restrictions on data access stemming from gaps in Italian legislation on data protection issues. This issue did not allow us to provide recent evidence, and we cannot rule out the possibility that including data of the year 2020 may have influenced some of the outcomes. Of note is that coronavirus disease 2019 (COVID-19) impacted on diagnostic delays of IBD (16, 27). Other strengths must also be considered. The use of administrative database allowed analyzing a large sample size. Consequently, the rigorous statistical method gave robustness to our findings. Another notable aspect of our analysis is the comparison between short-term and long-term possible missed diagnoses. Although both subgroups exhibited increased healthcare utilization compared to patients with a timely diagnosis, the risk profiles were slightly different. Patients with a short-term missed diagnosis showed a higher probability to access healthcare facilities compared with those possibly having a long-term missed diagnosis, suggesting that an imminent diagnostic clarification may still be preceded by a period of clinical uncertainty and exacerbated symptoms. In contrast, those with long-term missed diagnoses appeared to accumulate a similar degree of acute care utilization over time, albeit with minor variations in the HR values. These patterns indicate that the duration of a diagnostic delay might not be linearly correlated with the severity of acute exacerbations, but does contribute to a sustained burden on tertiary care resources.

This study emphasizes the need for enhanced diagnostic strategies within tertiary care settings. Increased awareness and education among non-specialist physicians, particularly those working in EDs and primary care, could be pivotal in recognizing early or atypical presentations of UC. The implementation of refined screening protocols and the integration of multidisciplinary teams may also help in reducing diagnostic delays. Our study provides compelling evidence that missed diagnoses in UC, even when they do not affect drug utilization, are associated with a significant increase in acute healthcare utilization. This underscores the clinical and economic importance of a timely diagnosis of UC. The comparison with Crohn’s disease further highlights that the adverse consequences of a delay are a common thread, although the diagnostic challenges may differ between these two IBD subtypes. Avoiding a missed diagnosis in UC may lead to improved patient outcomes, more efficient use of healthcare resources, and a better overall understanding of the natural history of the disease.

## Data Availability

The anonymized data of the dataset are managed by the Agenzia Regionale di Sanità Toscana and all relevant data is contained within the article in the **Supplementary Material**, further inquiries can be directed to the corresponding author/s. All anonymized data are used within Articles 110 (as amended by Law no. 56 of 2024) and 110-bis of Legislative Decree no. 196 of 2003 (Privacy Code), as well as by GDPR Provision no. 298 of 9 May 2024 (Provision). Requests to access these datasets should be directed to the corresponding author.
