# Supplementary material for: Possible underdiagnosis of ulcerative colitis in tertiary care can affect medication use and access to healthcare facilities
Source: Front Immunol. 2026 Feb 10;17:1693891. doi: 10.3389/fimmu.2026.1693891 (PMC12929424; doi:10.3389/fimmu.2026.1693891)
Supplement: Supplementary file 1 [file DataSheet1.docx]

Supplementary Material

# Supplementary Data

STROBE Statement—Checklist of items that should be included in reports of ***cohort studies***

|  | Item No | Recommendation |
| --- | --- | --- |
| **Title and abstract** | 1 | Pages 1-2 |
|  |  | Page 2 |
| Introduction | | |
| Background/rationale | 2 | Pages 2-3 |
| Objectives | 3 | Page 3 |
| Methods | | |
| Study design | 4 | Page 3 |
| Setting | 5 | Page 3 |
| Participants | 6 | Pages 3-4 |
|  |  | na |
| Variables | 7 | Page 4 |
| Data sources/ measurement | 8* | Pages 3-5 |
| Bias | 9 | Pages 4-5 |
| Study size | 10 | na |
| Quantitative variables | 11 | Pages 4-5 |
| Statistical methods | 12 | Page 4-5 |
|  |  | Page 5 |
|  |  | na |
|  |  | na |
|  |  | na |
| Results | | |
| Participants | 13* | Page 5 |
|  |  | Na |
|  |  | Figure 1 |
| Descriptive data | 14* | Pages 5-6 |
|  |  | Na |
|  |  | na |
| Outcome data | 15* | Pages 5-6 |
| Main results | 16 | Pages 5-6 |
|  |  | Na |
|  |  | Na |
| Other analyses | 17 | Pages 5-6 |
| Discussion | | |
| Key results | 18 | Page 6 |
| Limitations | 19 | Page 9 |
| Interpretation | 20 | Pages 6-9 |
| Generalisability | 21 | Pages 9-10 |
| Other information | | |
| Funding | 22 | Page 10 |

*Give information separately for exposed and unexposed groups.

**Note:** An Explanation and Elaboration article discusses each checklist item and gives methodological background and published examples of transparent reporting. The STROBE checklist is best used in conjunction with this article (freely available on the Web sites of PLoS Medicine at http://www.plosmedicine.org/, Annals of Internal Medicine at http://www.annals.org/, and Epidemiology at http://www.epidem.com/). Information on the STROBE Initiative is available at http://www.strobe-statement.org.

# Supplementary Figures and Tables

***Box 1.*** *ICD-9 (International Classification of Diseases, 9^th^ edition) codes used for the extraction of patients with ulcerative colitis.*

| **Disease** | **ICD-9 code** | **Exemption code** |
| --- | --- | --- |
| Ulcerative colitis | 556* | 009.556 |
| Ulcerative enterocolitis (chronic) | 556.0 |  |
| Ulcerative ileocolitis (chronic) | 556.1 |  |
| Ulcerative proctitis (chronic) | 556.2 |  |
| Ulcerative proctosigmoiditis (chronic) | 556.3 |  |
| Colon pseudopoliposis | 556.4 |  |
| Left-sided ulcerative colitis (chronic) | 556.5 |  |
| Ulcerative pancolitis | 556.6 |  |
| Other ulcerative colitis | 556.8 |  |
| Ulcerative colitis | 556.9 |  |

***Box 2.*** *ICD-9 (International Classification of Diseases, 9^th^ edition) codes used for the extraction of accesses to Emergency department (ED) and hospitalizations. The codes identify gastrointestinal events for the detection of symptoms of ulcerative colitis.*

| **Event** | **ICD-9 code** |
| --- | --- |
| Abdominal pain | 789.0* |
| Seat abdominal pain not specified | 789.00 |
| Abdominal pain of the upper right quadrant | 789.01 |
| Abdominal pain of the upper left quadrant | 789.02 |
| Abdominal pain of the lower right quadrant | 789.03 |
| Abdominal pain of the lower left quadrant | 789.04 |
| Periumbilical abdominal pain | 789.05 |
| Epigastric abdominal pain | 789.06 |
| Generalized abdominal pain | 789.07 |
| Abdominal pain of other specified locations | 789.09 |
| Diarrhoea | 787.91 |
| Faecal incontinence, faecal urgency | 787.6 |
| Faecal mucus | 792.1 |
| Hyperperistalsis | 787.4 |
| Proctitis | 569.49 |
| Rectal tenesmus | 787.99 |
| Rectorrhagia/haematochezia/melena | 578.1 |

***Box 3.*** *ATC code for azathioprine and non-conventional therapy drugs*

| **Drug** | **ATC** |
| --- | --- |
| azathioprine | L04AX01 |
| infliximab | L04AB02 |
| adalimumab | L04AB04 |
| golimumab | L04AB06 |
| vedolizumab | L04AA33 |
| tofacitinib | L04AA29 |
| ustekinumab | L04AC05 |

***Box 4.*** *Algorithm for the infusion procedure.*

| **Procedure** | **Algorithm** |
| --- | --- |
| Infusion of immunosuppressant drug | Specialist procedure code 99.24.2 AND specialist therapeutic code 058 |

***Box 5.*** *ICD-9 (International Classification of Diseases, 9^th^ edition) codes used for the extraction of surgery procedures.*

| **Surgery** | **ICD-9 code** |
| --- | --- |
| Colectomy (partial) (segmental) (subtotal); enterocolectomy NIA | 45.79 |
| Multiple segmental | 45.71 |
| Cecum (with terminal ileum) | 45.72 |
| Right (radical); right emicolectomy (extended) | 45.73 |
| Transverse | 45.74 |
| Left (Hartmann) (inferior) (radical); left emicolectomy | 45.75 |
| Sigmoid | 45.76 |
| Total | 45.8 |
| Ileostomy | 46.20 |
| With loop | 46.01 |
| Temporary; Tangential (temporary);  Tube (temporary); Of Hendon (temporary); Of Paul (temporary) | 46.21 |
| Continent (permanent) | 46.22 |
| Permanent; with transplant in new location | 46.23 |
| With delayed opening | 46.24 |
| Removal, ileostomy | 45.33 |
| Cecum-ileostomy | 45.93 |
| Ileostomy (manual) | 96.28 |
| Ileo pouch-anal anastomosis | 45.95 |

***Box 6:*** *Algorithms for the detection of gastroenterological visits.*

| **Procedure** | **Algorithm** |
| --- | --- |
| Gastroenterological visit | ICD-9 code 89.7 AND specialist therapeutic code 058 |
|  | ICD-9 code 89.1 AND specialist therapeutic code 058 |
|  | Specialist procedure code 99.24.2 AND specialist therapeutic code 058 |

***Table S1.*** *Patients’ characteristics at cohort entry. The table shows data on patients grouped according to the criteria defining the index date.*

| **Patients** | **Overall** | **Diagnosis*** | **UC co-payment exemption** |
| --- | --- | --- | --- |
| Number, n (%) | 3,804 | 2,168 | 1,636 |
| Age, mean (SD) | 53.38 (18.7) | 59.23 (19.3) | 45.62 (14.7) |
| Female, n (%) | 1,803 (47.4) | 1,063 (59.0) | 740 (41.0) |
| Concomitant drugs, mean (SD) | 0.31 (1.0) | 0.39 (1.2) | 0.21 (0.8) |
| ***:** UC diagnosis detected from Hospital discharge forms or Emergency department accesses; n: number; SD: standard deviation. | | | |

## Supplementary Figures

***Figure S1:*** *Kaplan-Meier curves for azathioprine and non-conventional therapy outcomes computed for the categorical variables of missed diagnosis.*


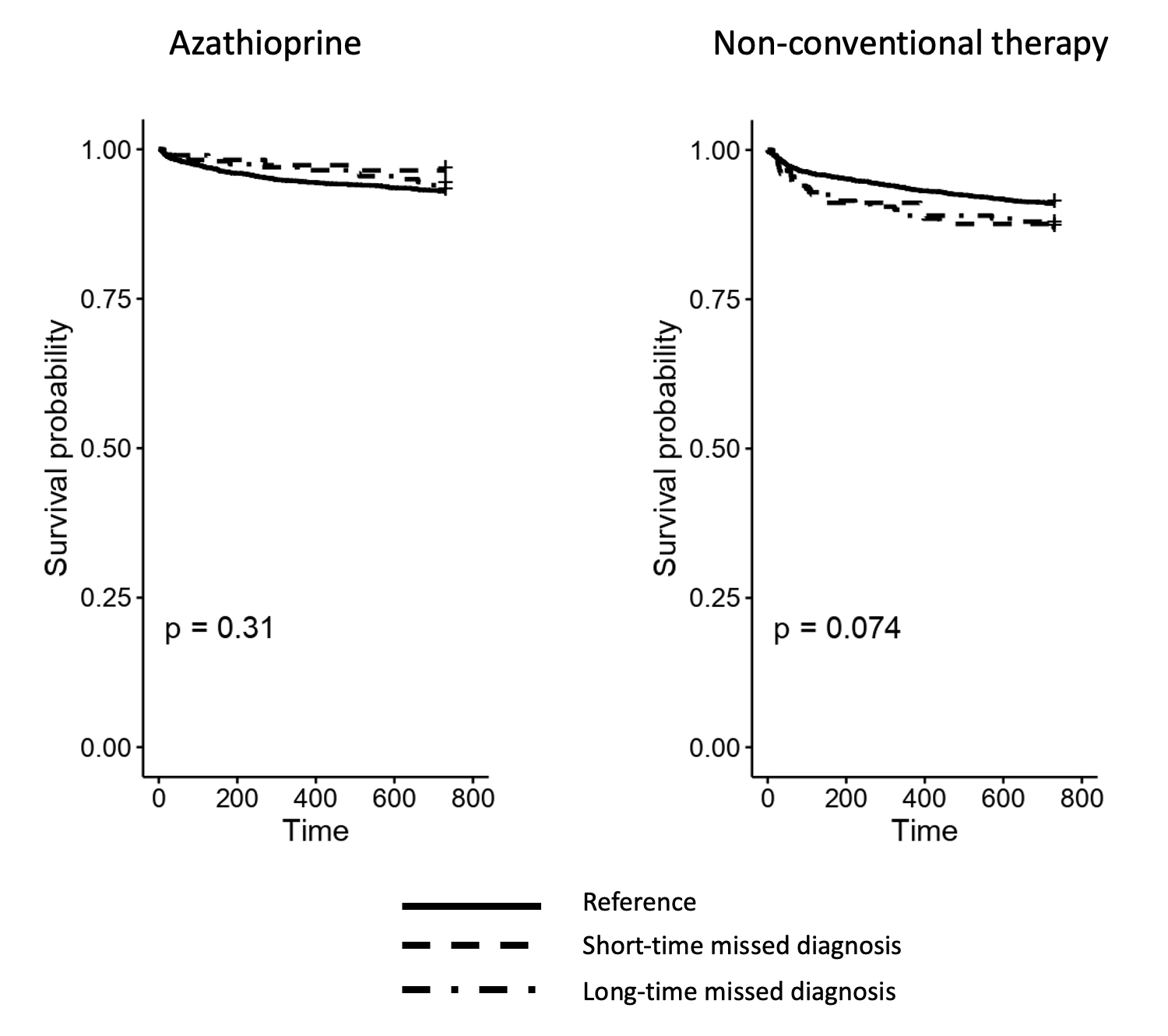


***Figure S2:*** *Kaplan-Meier curves for surgery computed for the dichotomous (A) and categorical (B) variables of missed diagnosis.*


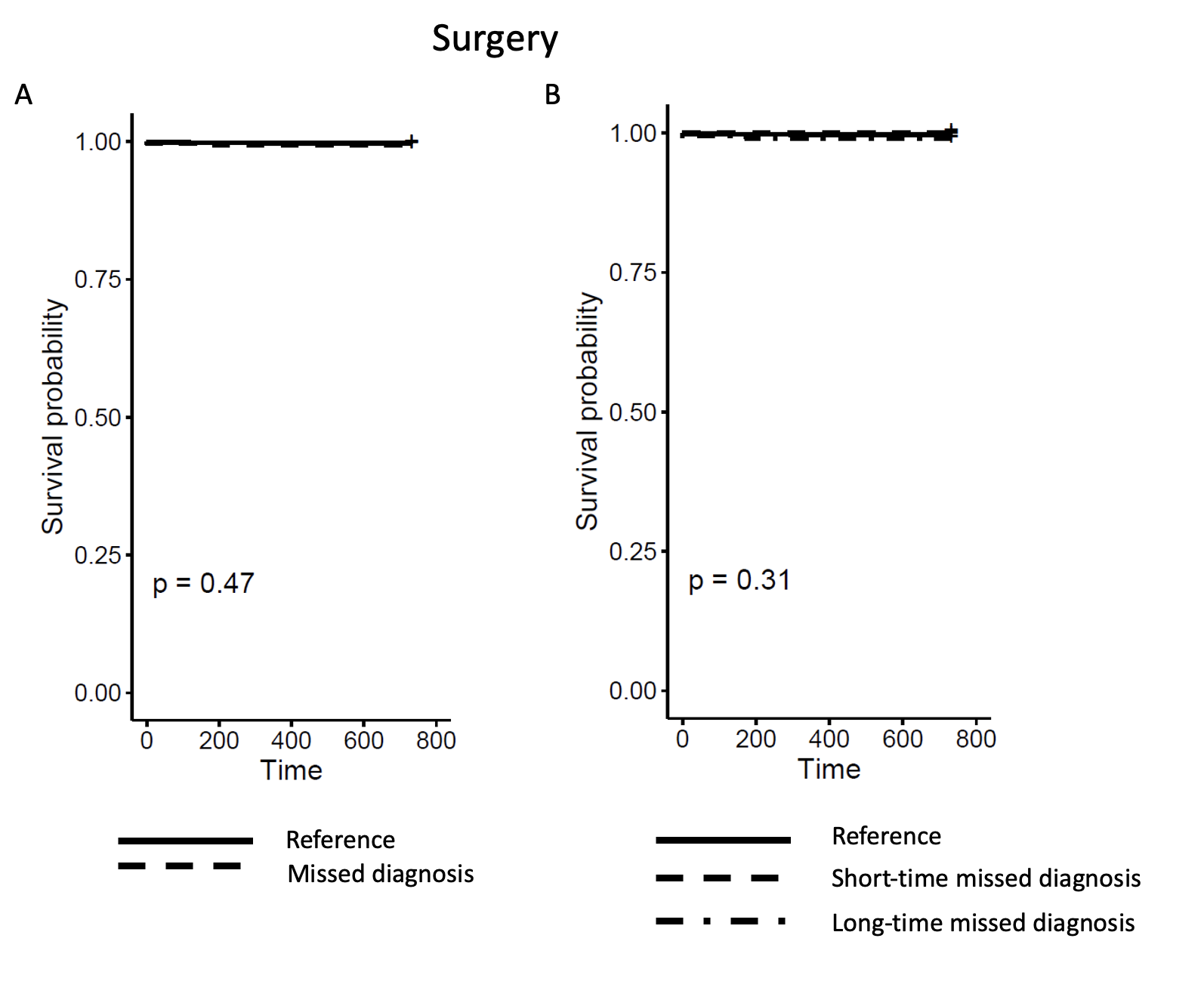


**
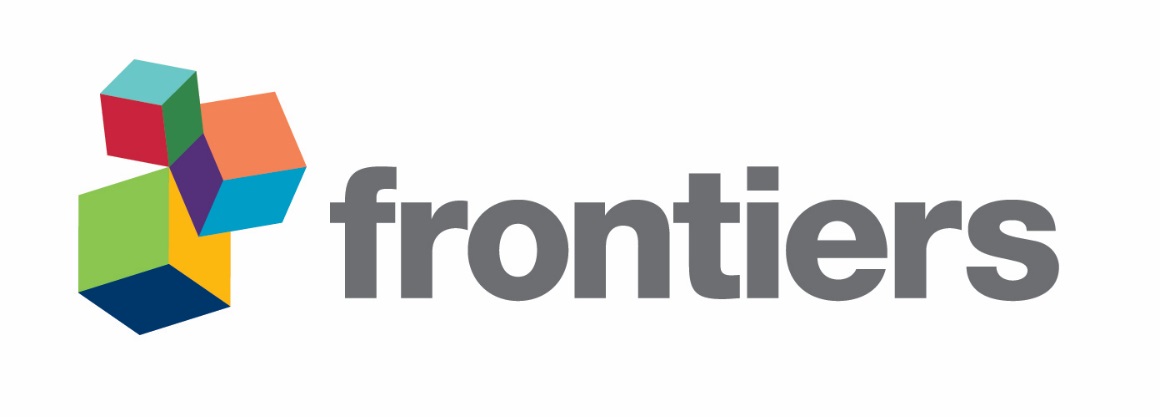
**
